# Modern Approaches to the Genome Editing of Antibiotic Biosynthetic Clusters in Actinomycetes

**DOI:** 10.32607/actanaturae.23426

**Published:** 2023

**Authors:** J. A. Buyuklyan, Yu. V. Zakalyukina, I. A. Osterman, M. V. Biryukov

**Affiliations:** Center for Translational Medicine, Sirius University of Science and Technology, Sochi, 354340 Russian Federation; Lomonosov Moscow State University, Moscow, 119234 Russian Federation; Skolkovo Institute of Science and Technology, Skolkovo, Moscow Region, 143025 Russian Federation

**Keywords:** antibiotic biosynthetic clusters, genome editing, site-directed mutagenesis, actinomycetes, antibiotics

## Abstract

Representatives of the phylum *Actinomycetota *are one of the main sources of secondary metabolites, including antibiotics of various classes. Modern studies using high-throughput sequencing techniques enable the detection of dozens of potential antibiotic biosynthetic genome clusters in many actinomycetes; however, under laboratory conditions, production of secondary metabolites amounts to less than 5% of the total coding potential of producer strains. However, many of these antibiotics have already been described. There is a continuous “rediscovery” of known antibiotics, and new molecules become almost invisible against the general background. The established approaches aimed at increasing the production of novel antibiotics include: selection of optimal cultivation conditions by modifying the composition of nutrient media; co-cultivation methods; microfluidics, and the use of various transcription factors to activate silent genes. Unfortunately, these tools are non-universal for various actinomycete strains, stochastic in nature, and therefore do not always lead to success. The use of genetic engineering technologies is much more efficient, because they allow for a directed and controlled change in the production of target metabolites. One example of such technologies is mutagenesis-based genome editing of antibiotic biosynthetic clusters. This targeted approach allows one to alter gene expression, suppressing the production of previously characterized molecules, and thereby promoting the synthesis of other unknown antibiotic variants. In addition, mutagenesis techniques can be successfully applied both to new producer strains and to the genes of known isolates to identify new compounds.

## INTRODUCTION

*Actinomycetota *phylum members, high G–C content Gram-positive bacteria, are one of the main sources of biologically active substances [1, 2]. Modern high-throughput sequencing techniques enable the detection of dozens of biosynthetic clusters of potential antibiotics in the genomes of many actinomycetes [3]; however, the production of secondary metabolites using traditional laboratory screening techniques [4, 5], which were pioneered by Waksman in the 1940s, amounts to less than 5% of the full genetic potential of the producer strains [6, 7]. Often, these antibiotics have already been described. Some known antibiotics are frequently “rediscovered,” whereas novel molecules may remain virtually invisible against the general background. The usual approaches to increase the production of novel antibiotics include the creation of optimal cultivation conditions by modifying the growth medium composition [8], co-cultivation methods [9], microfluidics methods [10], and the use of various transcription factors to activate silent genes [11, 12]. Unfortunately, these tools are non-universal for various actinomycete strains, stochastic in nature, and, therefore, they are not always success ful. Genetic engineering technologies are much more effective, because they provide for targeted and controllable changes in the production of target metabolites [13]. One of these technologies is mutagenesis- based genome editing of antibiotic biosynthetic clusters [14 , 15, 16]. This targeted approach can alter gene expression [17] and inhibit the production of already characterized molecules, thereby facilitating the synthesis of heretofore unknown antibiotics. In addition, mutagenesis techniques can be successfully used in both new producer strains and the genes of known isolates in order to identify novel compounds.

## GENOME OF ACTINOMYCETES

The genome of actinomycetes is represented by a 5- to 10-Mb circular or linear DNA molecule (*Streptomyces* spp.) with high G–C content amounting to more than 70% in some genera [18, 19, 20]. In actinomycetes— representatives of prokaryotes—implementation of genetic information, namely transcription and translation, is coupled in time and space due to the lack of internal compartmentalization of the cell [21]. The ribosome can bind to a RNA polymerase- synthesized mRNA and begin protein synthesis. In actinomycete genomes, genes encoding bioactive compounds are usually organized into biosynthetic gene clusters (BGCs) [22, 23]. BGCs are a group of two or more genes that share a common transcription start point and together encode a biosynthetic pathway for the production of a specialized metabolite. These genes contain information about the regulatory proteins that control the timing and level of expression and secretion of a particular metabolite.

**Fig. 1:**
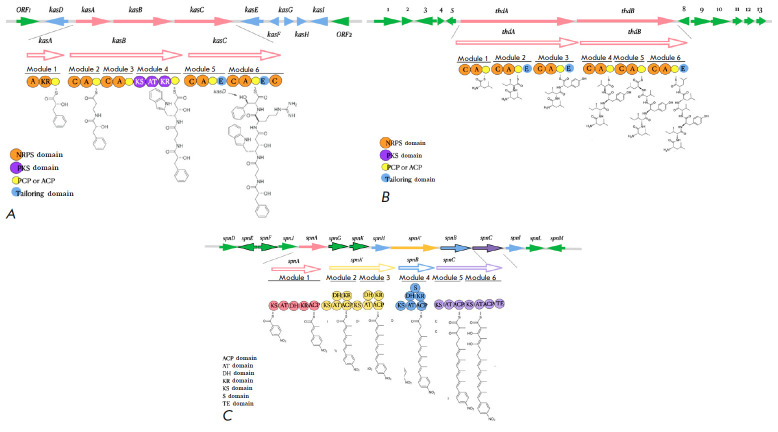
Schematic representation of antibiotic biosynthesis clusters: (*A*) thermoactinoamide A from *Thermoactinomyces* sp. [28]; (*B*) kasugamycin from *Streptomyces kasugaensis *[29]; (*C*) spinosyn from *Streptomyces albus J1074 *[30]

There are different structural BGC classes, including non-ribosomal peptide synthetases (NRPSs), polyketide synthetases (PKSs), terpenes, and bacteriocins [24]. NRPSs and PKSs are common markers for the detection of secondary metabolites, because they synthesize structurally diverse molecules exhibiting antibiotic and immunosuppressive properties, as well as great pharmaceutical potential [25, 26]. These regions can be used to identify new antibiotic biosynthetic pathways [27] (*Fig. 1*).

According to bioinformatics data generated by the DOE Joint Genome Institute, all antibiotic producers contain dozens of potential biosynthetic clusters; i.e., they have much greater biosynthetic potential compared with that identified by routine cultivation [27].

Currently, there are various approaches to activating silent clusters [31]. They may be divided into two groups: the first group is based on the heterologous expression of clusters in model *Escherichia coli *or *Saccharomyces cerevisiae *strains [32, 33], and the second uses genome editing directly in the producer strains [34, 35]. Each of these approaches has its own advantages and disadvantages. In the case of heterologous expression of clusters in *E. coli *or* S. cerevisiae *strains [36], the advantages are as follows: the simplicity associated with the transformation and expression of genes in well-studied model microorganisms, which provides a means to regulate the expression level of the antibiotic synthesis genes. This control of gene expression regulation may be implemented by means of inducible or constitutive promoters. Therefore, specific metabolites would be synthesized either in the presence of inducer molecules or permanently in a heterologous strain. In addition, model organisms, in particular *Escherichia coli*, are free of endogenous secondary metabolic pathways, which allows to obviate the influence on target cluster synthesis. Despite the positive aspects of this approach, there are a number of limiting factors: first, cluster transfer is based on homologous recombination [37], whose accuracy decreases as the number of events increases. Second, there are differences in the nucleotide sequence of the triplets encoding amino acids in different organisms. This leads to an additional step associated with the generation of a codonoptimized sequence for the synthesis of the target antibiotic molecule. These manipulations are necessary to eliminate frameshifting between native strains and hosts. In addition, some techniques require their own consensus sequences, such as attP-attachment sites that mediate site-specific recombination [38], and special plasmids, which makes the procedure more complex and labor-intensive [39].

An alternative approach to activating silent clusters is genome editing directly in the producer strains. This approach introduces mutations in the original wild-type strain and controls changes directly in it [40]. These genetic manipulations enable to study the effect of a specific mutation on other metabolic pathways not involved in the biosynthesis of a particular metabolite [41]. Of course, this technique has its own disadvantages, but there are ways to avoid them, and we will discuss them below.

## APPROACHES TO GENOME EDITING IN ACTINOMYCETES

Compared to “traditional” targets for genetic modification, such as *E. coli* and *S. cerevisiae* [42], actinomycetes have a complex regulatory apparatus that prevents effective, targeted transformation of their genome [43, 44]. Nonetheless, there are approaches to introducing point mutations into the genetic apparatus of producer strains. All genome editing techniques may be divided into two categories: spontaneous [45] and site-directed mutagenesis [46].

**Spontaneous mutagenesis**

**Fig. 2:**
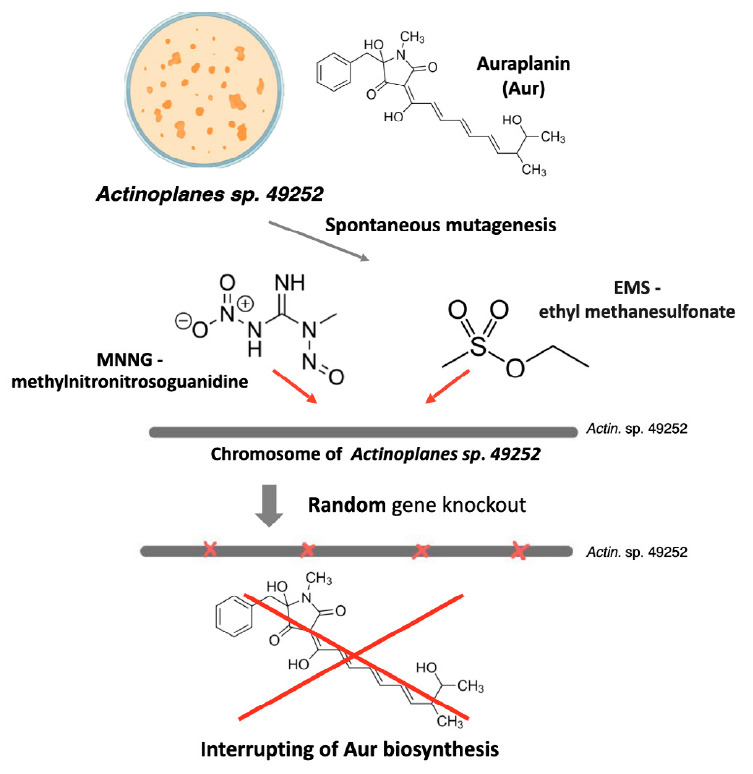
Schematic representation of mutations introduced into the genome of actinomycetes using spontaneous chemical mutagenesis

Spontaneous mutagenesis is associated with the introduction of random point mutations into DNA using a mutagen. This approach is used to solve several problems: introduction of single-nucleotide substitutions to produce new biosynthetic products [47]; an auxiliary tool for clarifying the nucleotide sequence of antibiotic biosynthetic clusters [48]. The mutagens used are methylnitronitrosoguanidine (MNNG) [49] that adds alkyl groups to the O6 of guanine and O4 of thymine, which leads to transition mutations between the GC and AT pairs [50], and ethyl methane sulfonate (EMS) that causes transition mutations between the GC and AT pairs [51] (*Fig. 2*). In addition to transitions of the purine and pyrimidine bases, a mutagen can also change the expression level of specific genes [52]. Because these single nucleotide substitutions are introduced randomly, alkylation/methylation of nitrogenous bases occurs in different regions of the genome. For example, modification of the promoter (regulatory region) nucleotide sequence can suppress the expression of biosynthetic gene clusters [51], and mutations in a BGC coding region can produce other genetic products and, as a consequence, new substances [48, 53].

Thus, spontaneous mutagenesis helps solve some of the problems associated with the search for new molecules, but this process is probabilistic in nature and does not guarantee reproducibility of the results; so, it cannot be used to develop a full-fledged technique for producing new antibiotics.

**Site-directed mutagenesis**

As mentioned above, actinomycetes implement only a small part of their biosynthetic activity and one antibiotic, such as streptothricin, can be found in every tenth isolate, while others, such as tetracycline and actinomycin D, are found at a rate of one per 100–1,000 isolates [1]. To produce novel antibiotics and their modifications using a traditional approach, such as a Waksman platform [54], it is necessary to test tens of millions of isolates. This routine approach is labor-, time-, and resources-intensive. Importantly, even known strains are a source of a huge variety of molecules with antibacterial activity [55] whose gene expression is masked by predominantly detected, known antibiotics [1].

Culp et al. proposed a concept based on the idea that disruption of the conserved biosynthetic genes of known antibiotics produced by strains may facilitate the discovery of novel metabolites whose activity has not yet been detected [56, 57]. This problem is solved using various genetic engineering tools aimed mainly at introducing deletions into the biosynthetic gene clusters of the producer strains. All these techniques may be divided into three large categories differing in their driving molecular mechanism.

**Fig. 3:**
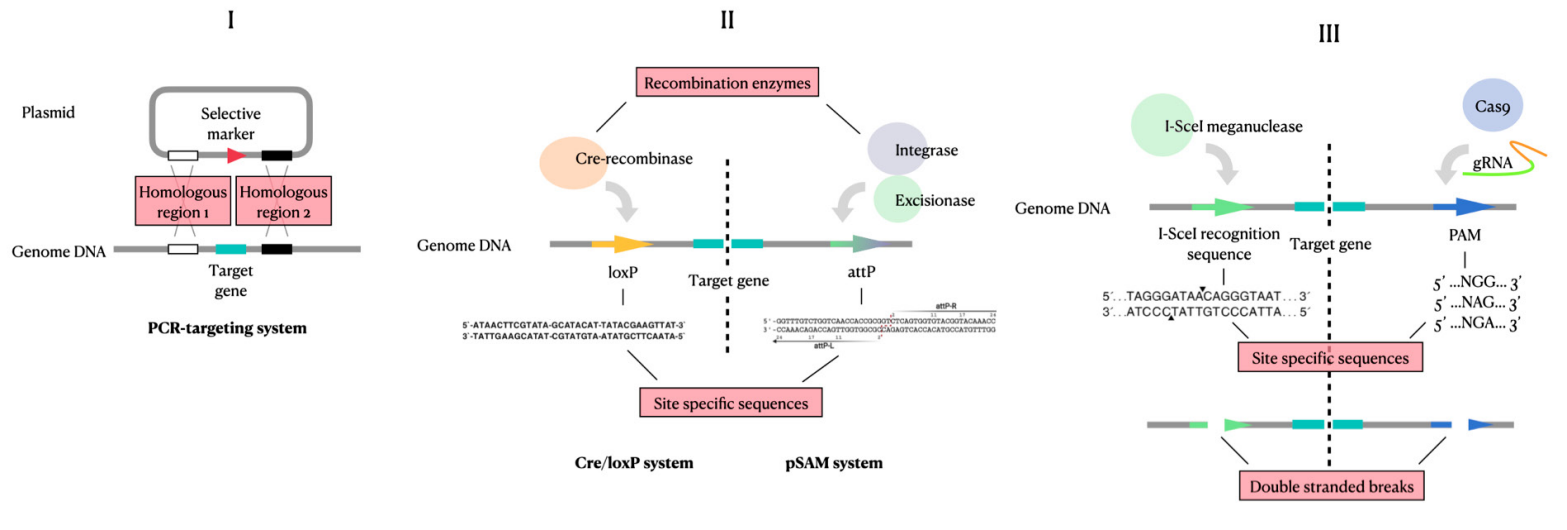
Schematic representation of the molecular mechanisms underlying genetic engineering techniques for introducing mutations into the cell genome. Process I – homologous recombination; process II – site-specific recombination; process III – nuclease-induced double-strand breaks, followed by their repair

Three fundamental processes are used as tools to introduce mutations: homologous recombination providing the basis for the PCR-targeting system that uses the homologous sequences required for recombination to produce deletions. The second molecular mechanism is site-specific recombination, used in the Cre-loxP recombination system and pSAM2 sitespecific recombination system. The key feature is the presence of special sites: the loxP sequence for Cre recombinase and the attP sequence for the pSAM2 system. This process involves not only specific sequences, but also enzymes that perform recombination in strictly defined regions of the genome, which increases the accuracy of the process. The third process underlying site-directed mutagenesis is the introduction of double-strand breaks by nucleases, such as I-SceI meganuclease (I-SceI meganuclease-promoted recombination system) and Cas-nickase (CRISPR/Casbased genome editing). Double-strand breaks introduced into DNA are subsequently recovered by cell repair systems (*Fig. 3*).

## RECOMBINATION-BASED GENOME EDITING

**PCR-Targeting System**

The first-ever genome editing system, developed for* E. coli *cells, is based on homologous recombination using the λ-Red system [58]. Homologous recombination [59] is a widespread biological phenomenon that occurs in the cells of living organisms. This process is highly conserved and involves breakage and repair of double-stranded DNA (dsDNA) [60, 61]. In addition, homologous recombination is a tool for introducing point mutations into the bacterial genome [62]. This process provided the basis for developing a PCR-mediated genome editing tool that replaces the target sequence in the cell genome with an amplified fragment of the selective marker gene (*Table 1*).

**Fig. 4:**
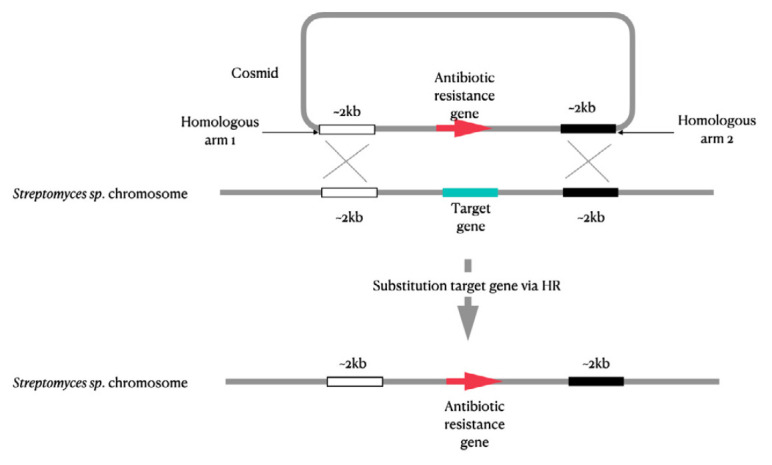
Schematic representation of mutations introduced into the genome of actinomycetes using PCR-mediated genome editing

For a successful homologous recombination, 2 Kb flanking sequences are required. A deletion was for the first time introduced into the geosmin biosynthetic gene cluster of *St. coelicolor A3(2)* using a PCRmediated technique [66, 68] (*Fig. 4*).

**Table 1:** Selective markers for the genetic engineering of actinomycetes

No	Resistance gene	Resistance	Antibiotic	Plasmid
1.	aac(3)IV – aminoglycoside N(3)-acetyltransferase	Resistance to antibiotics comprising a 2-deoxy-streptamine ring	Apramycin	pCRISPomyces [63]; pStreptoBAC V [1]
2.	aph(3)II – aminoglycoside modifying enzyme	Resistance to aminoglycoside antibiotics	Kanamycin A and B, neomycin B and C	pCAP01 [64]; pESAC13 [65]
3.	aadA – aminoglycoside (3’’) (9) adenylyltransferase	Resistance to streptogramins and aminoglycosides	Streptomycin, spectinomycin	pIJ778 [66]
4.	vph – phosphotransferase	Viomycin resistance	Viomycin	pIJ780 [66]
5.	ermE – methyltransferase	- erythromycin resistance	gene	Resistance to macrolide antibiotics Erythromycin pBF24 [67]
6.	hyhB – hygromycin resistance gene	Resistance to aminoglycoside antibiotics	Hygromycin B	pBF27 N [67].

This controlled genetic engineering enables one to generate antibiotics through combinatorial biosynthesis in the producer strain. The strain is depleted of genes of the main endogenous secondary metabolites (avermectin and filipin in *St. avermitilis*), transposon genes [69], and the IS sequences [70] that do not affect the strain growth rate but promote genome stability.

Despite successful results [66, 68], there remains limitations in PCR-mediated genome editing due to its non-universality for different actinomycete strains.

**Cre-loxP recombination system**

**Fig. 5:**
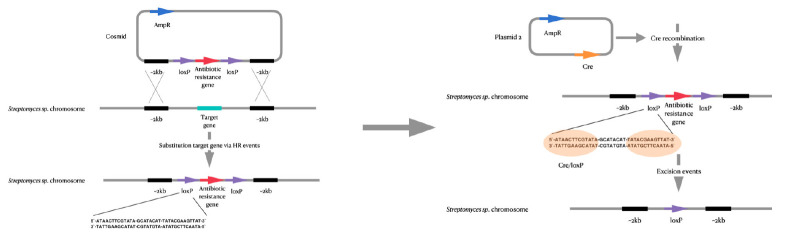
Schematic representation of mutations introduced into the genome of actinomycetes using Cre/loxP-mediated genome editing

Cre-loxP recombination is used to make large deletions in the genome of bacterial cells [71, 72] using Cre recombinase [73, 74]. To introduce a mutation, two loxP (locus of crossing (x) over, P1) sequences flanking the target gene are required for site-specific Cre recombinase-mediated recombination (*Fig. 5*) [75].

The mechanism for introducing mutations involves successive recombination stages. First, two loxP sequences are introduced into the actinomycete genome in such a way as to flank the target gene. This process is mediated by two homologous recombination events [68]. Next, the Cre protein gene is expressed and the recombinase recognizes the introduced loxP sequences and performs site-specific recombination [60], leading to the deletion of the target gene. After completing the process, one of the loxP sequences is retained in the actinomycete genome.

This technique was used to produce a 1.4-Mb deletion in the geosmin biosynthetic gene cluster in the* St. avermitilis *genome [68]. This technique is more accurate than the PCR-mediated approach where recombination is controlled by the cell’s internal ma chinery and occurs at the homologous flanking regions of the target gene [59], which is probabilistic in nature. The specificity and accuracy of the Cre/loxP approach are based on the presence of loxP flanking sequences that are specifically recognized by Cre recombinase [76]. Furthermore, the *cre *gene sequence is controlled by an inducible promoter in a separate plasmid, which provides control over the Cre recombinase expression [77]. The drawback of this system is the preservation of loxP fragments in the genome with changes in the genomic content, in addition to the target mutation–deletion.

**pSAM2 site-specific recombination system**

The pSAM2 system [78], like the Cre-loxP approach, is based on site-specific recombination [79]. But in this case, the specificity is associated not with recombinase activity, but with certain sequences in the genome— attachment sites attP (pSAM2 plasmid) and attB (genomic DNA of the bacterium) [80, 81, 82]. These attB sites, encoded by the non-replicative pSAM2 plasmid, are introduced into the genome of actinomycetes through homologous recombination [59]. It is noteworthy that after removal of selective pressure, the plasmid is eliminated from actinomycete cells. The Att sites introduced into the genomic sequence flank the target gene on both sides.

**Fig. 6:**
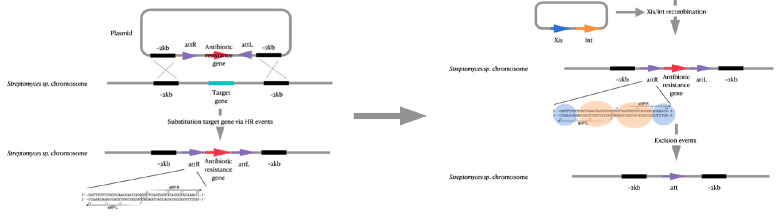
Schematic representation of mutations introduced into the genome of actinomycetes using pSAM-mediated genome editing

The introduction of a mutation using the pSAM2- based system includes the following steps: at the first step, recombination occurs at the attP/attB sites, which is accompanied by plasmid integration into the actinomycete genome. Next, the target gene is deleted by Xis excisionase. The Xis protein gene is located in a self-replicating plasmid and is eliminated from actinomycete cells when selective pressure decreases [83, 84] (*Fig. 6*).

This approach was used to delete a 90 Kb rifampicin biosynthetic cluster in *A. mediterranei DSM 40773* cells [78]. The main advantage of this technique is that mutant strains contain additional 30–40 bp inserts in the genomic sequence, which does not affect the reading frame [85].

## NUCLEASE-BASED GENOME EDITING

**I-SceI meganuclease-promoted recombination system**

The next genome editing technique is based on the introduction of double-strand breaks by the I-SceI meganuclease-promoted recombination system [86]. In this case, I-SceI meganuclease recognizes a unique 18 bp sequence, introduces a double-strand break, and starts the recombination process [87]. I-SceI meganuclease was first found in *S. cerevisiae *mitochondria [88].

**Fig. 7:**
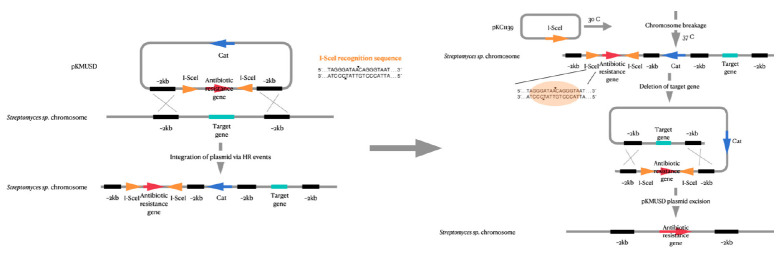
Schematic representation of mutations introduced into the genome of actinomycetes using I-SceI-mediated genome editing

In practice, a codon-optimized sequence of the I-SceI meganuclease gene [89, 90] and the temperature- sensitive plasmid pHZ1358 and its derivatives (pKC1139 and pJTU1278) are required to introduce deletions or substitutions into the nucleotide sequence of actinomycete strains (*Fig. 7A*). In addition, insertion of 18 bp into the producer strain genome is required. This technique was used to delete the actinorhodin (*Act*) gene from *St. coelicolor A3(2) *cells [86, 91].

The process includes a series of homologous recombination events necessary to introduce an 18-nucleotide I-SceI target sequence into the actinomycete genome. These sites are encoded by the self-replicating plasmid pKMUSD. Next, the I-SceI protein gene is expressed under the control of the inducible tipA promoter [92]. Meganuclease recognizes a specific sequence in the actinomycete genome and introduces double-strand breaks that are then repaired using homologous fragments present in the cell genome [86]. It is worth noting that the I-SceI protein gene is localized in the temperature-sensitive plasmid pKC1139; therefore, after the second homologous recombination event, as the temperature rises to 36°C, the plasmid, together with the I-SceI protein gene, is eliminated [93]. This activity is controlled by the temperature-sensitive origin of pSG5 replication in the plasmid [86, 94]. This enables the introduction of deletions without additional changes in the genomic content (*Table 2*).

**Table 2:** The site-directed mutagenesis techniques used in actinomycetes

No	Technique	Advantages	Disadvantages	Efficiency
1.	PCR-Targeting System	No additional tools, except PCR, are needed to introduce mutations.	Complex protocols, time-consuming procedures, universality for different actinomycete strains; deletion is accompanied by introduction of a selective marker into the genome.	Efficacy was shown only in the geosmin BGC of a model St. coelicolor strain.
2.	Cre-loxP Recombination System	Opportunity to delete large gene regions of about 1.4 Mb. [16]. Greater specificity due to Cre recombinase.	Time-consuming procedures, changes in genomic content, apart from the target mutation (deletion), due to preservation of loxP fragments.	A positive result was shown only for the geosmin BGC in a model St. avermitilis strain.
3.	pSAM2 Site-Specific Recombination System	Deletion of entire BGCs, minimal changes in the reading frame after excision of the genetic construct.	Time-consuming procedures, preservation of small sequences in the bacterial genome.	The approach is effective not only in model streptomycete strains but also in rare genera such as Actinoplanes mediterranei. The technique was effective in 90 Kb antibiotic BGCs (rifampicin cluster).
4.	I-SceI Meganuclease-Promoted Recombination System	Implementation of deletions without additional changes in genomic content.	Genome editing requires a codon-optimized I-SceI meganuclease gene sequence and a temperature-sensitive plasmid pKC1139.	Efficiency was shown in the actinorhodin BGC of a model St. coelicolor strain.
5.	Cas9-Based Genome Editing	Opportunity to introduce genomic deletions up to 30 Kb [16]; opportunity to introduce mutations into the promoter sequence.	Toxicity of the Cas9 protein due to the off-target effect; DSBs require a G-rich PAM sequence (5’-NGG-3’); Introduced DSBs cannot always be eliminated by intracellular repair systems.	Efficiency varies from 21 to 100% both in model streptomycete strains and in three members of rare genera [16]. Widely used for editing antibiotic BGCs of various lengths.
6.	Cpf1-Assisted Genome Editing	High specificity due to the need in a T-rich PAM sequence (5′-TTV-3) for introducing DSBs.	Introduced DSBs cannot always be eliminated by intracellular repair systems.	Efficiency of the system has been demonstrated in various actinomycete strains. The Gpf1 protein exhibits specificity for the T-rich PAM sequence, which reduces the off-target effect by 26%, thereby increasing the efficiency from 47 to 100% [44, 60].
7.	CRISPR-BEST (CRISPRBase Editing SysTem)	Genome editing does not require DSBs; point mutations are introduced to create a stop codon.		The technique is applicable both to model actinomycete strains, such as St. coelicolor, and to members of rare genera. This technique is relatively new and has been tested in known BGCs such as actinorhodin.

The major drawback of the I-SceI meganucleasebased approach is a lack of the *tipA *gene for inducing nuclease genes in some strains. In addition, this process is accompanied by double-strand DNA breaks, so errors in the repair apparatus can lead to mutations not associated with the target deletion. The positive aspects of this technique include preservation of the genomic content without additional nucleotide sequences after the completion of genetic manipulations.

**CRISPR/Cas-based genome editing**

Technology based on the clustered, regularly interspaced short palindromic repeats CRISPR/Cas system, in particular the CRISPR/Cas9 system, has become a promising tool for the genetic engineering of actinomycete strains [95, 96, 97].

CRISPR/Cas is a natural system for defending prokaryotic cells against foreign DNA [98, 99, 100]. This technology is widely used for genome editing in organisms from various taxonomic groups. Unlike I-SceI meganuclease-based genome editing [101], the CRISPR/Cas-based technology does not require preintegration of a unique enzyme-recognized sequence into the target genome but uses a transcribed guide RNA (sgRNA, a chimera of crRNA and tracrRNA) or crRNA alone to selectively bind Cas proteins in any genomic region [102, 103]. The Cas9/crRNA/tracrRNA complex can target any DNA sequence, known as a protospacer, provided that its 3’-end carries an appropriate trinucleotide protospacer adjacent motif (PAM) [104, 105], such as NGG (N is any nucleotide) in *Streptococcus pyogenes *[106].

**Fig. 8:**
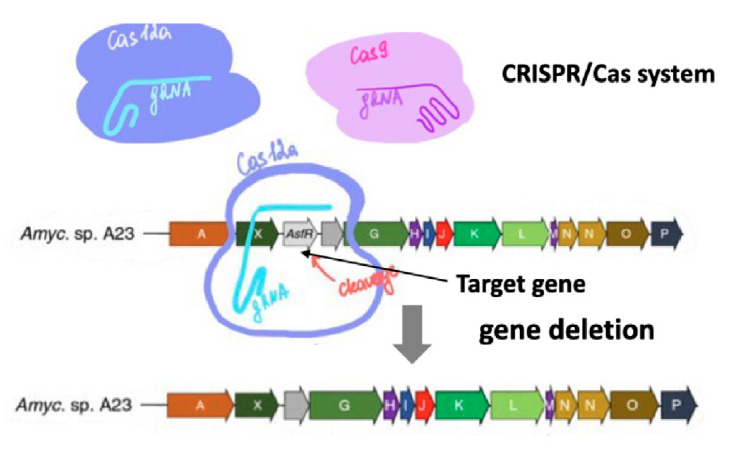
Schematic representation of mutations introduced into the genome of actinomycetes using CRISPR-Casmediated genome editing

The genome of streptomycetes is mainly edited with two Cas nucleases: the class 2 type II Cas9 from* Str. pyogenes *[107] and the class 2 type V Cpf1, also known as Cas12a, from *Francisella novicida* (*Fig. 8*) [103, 108, 109].

Compared with other genome editing technologies, the CRISPR/Cas system has clear advantages: high efficiency, ease of use, and rapid results [110].

*Cas9-based genome editing. *Based on the CIRSPR/ Cas9 system, two plasmid versions have been developed for manipulating the genome of streptomycetes: pCRISPomyces-1 and pCRISPomyces-2 [63].

pCRISPomyces-1 comprises crRNA and tracrRNA gene sequences and the *cas9 *gene. pCRISPomyces-2 includes the chimeric sgRNA cassette and *cas9* gene. Both plasmids use strong constitutive promoters for the expression of CRISPR/Cas elements and an optimized cas9 gene sequence for better expression in* Streptomyces *[111, 112].

Using this tool, Cobb et al. successfully achieved 20–31.4 Kb DNA deletions, including individual genes and clusters of antibiotic biosynthesis, with an efficiency of 21–100% in three different streptomycete species [63]. The introduction of such deletions into streptomycin and streptothricin biosynthesis clusters led to the identification of novel antibiotics in known producer strains: thiolactomycin, amicetin, phenanthroviridin, and 5-chloro-3-formylindole [113].

In addition to deletions, the CRISPR/Cas system allows for the introduction of mutations into promoters. Thus, it was possible to activate silent biosynthetic gene clusters of different classes in five *Streptomyces* strains and to identify unique metabolites, including a novel pentangular type II polyketide in *St. viridochromogenes* [114].

Despite the obvious advantages, the pCRISPomyces system has a number of disadvantages associated with the toxicity of the Cas9 protein to the bacterial cell. This is due to the cleavage of non-target DNA (off-target effect) [115, 116] and difficulty to use it in streptomycetes with a low DNA transformation efficiency [94]. Wang et al. developed a modified pWHU2653 plasmid-based CRSPR/Cas9 system with the Cas9 protein gene under the control of an inducible promoter, which provides the control over protein synthesis [117]. Also, double-strand break repair is ATP-dependent, so the *AtpD *gene encoding the β-subunit of ATP synthase was introduced into the pWHU2653 plasmid to enhance the editing efficiency (*Table 2*) [94].

*Cpf1-assisted genome editing**. ***Apart from the indicated disadvantages, the CRISPR/Cas9 system has a number of limitations. As mentioned earlier, the genome of actinomycetes has a high GC content [118], and recognition of the target sequence by the Cas9 protein requires a G-rich (PAM) sequence (5’-NGG-3′)’ e.g., 260 targets per 1,000 bp in *St. coelicolor *[119, 120]. The system, based on the Cas12a protein from* F. novicida, *gets around this limitation because double- strand breaks require a T-rich PAM sequence (5’-TTV-3) [121], which increases the specificity of the process [97]. Using Cpf1 nuclease, Yeo et al. achieved gene deletion in the 5-oxo-milbemycin A3/A4 producing strain *St. hygroscopicus *SIPI-KF, which could not be edited by Cas9 due to its high toxicity [120]. Thus, Cpf1 and alternative genome editing technologies complement current CRISPR/Cas-based tools and facilitate the discovery of novel biologically active substances in *Streptomyces *spp. and members of other actinomycete genera (*Table 2*) [93].

*CRISPR-BEST (CRISPR-Base Editing SysTem).* Nuclease-based genome editing techniques require the introduction of double-strand breaks in DNA, which may lead to genome instability due to failure of the repair systems [122]. David Liu developed an alternative CRISPR/Cas system-based technique that does not require DSBs. This technique generates point mutations leading to the emergence of a stop codon in the coding sequence [123]. The technique uses two types of deaminases: cytidine deaminase [124] converts cytosine to thymine, and adenine deaminase [125] leads to transitions, such as A–G and C–T. This difference was used to produce two genetic constructs: CRISPR-cBEST comprising a variant of the rat *APOBEC1 *cytidine deaminase gene (r*APOBEC1*) and CRISPR-aBEST with adenine deaminase controlled by the inducible tipA (*thiostrepton- responsive activator*) promoter [124]: so, the key factor is the presence of the *tipA *gene in the target strain [125]. In addition, both plasmids contain the Cas9 nickase gene [126] and a codon-optimized sgRNA sequence [103]. The use of these plasmids leads to the expression of deaminase genes and transitions. Deamination of adenine in an A : T pair or cytosine in a C : G pair results in the formation of new pairs, I : T and U : G, in one DNA strand. During replication, uracil in the new U : G pair is recognized as thymine and inosine in the I : T pair is recognized as guanine; this discrepancy leads to the activation of cell repair systems [96, 127].

In the first case, uracil DNA glycosylase (UDG) is activated [128], triggering the excision repair mechanism [129, 130], or the original pairs are repaired by the mismatch repair system [129, 131, 132]: thus, the original pairs are repaired, and DSBs are not required in further replication processes.

It should be noted that this system has shown good results in model *St. coelicolor *strains and in *St. griseofuscus* (*Table 2*).

## CONCLUSION

Genome mining and manipulations with the genome, in particular antibiotic gene clusters, represent an enormous potential in our efforts to identify new molecules that exhibit antibacterial activity. Importantly, the discovery of new BGCs in the genome of actinomycetes opens up broad opportunities for their editing; however, there are some limitations associated with these techniques and tools for changing the metabolic activity of strains.

Each of the described approaches can be used for specific genetic engineering tasks. For example, spontaneous mutagenesis is used as an additional tool to identify the BGC of a potential novel antibiotic. The introduction of random mutations into the genome of the producer strain may change the biosynthetic activity of a test metabolite, and further genomic analysis identifies the mutated gene region in the biosynthetic cluster. The key advantages of site-directed mutagenesis include its target specificity and efficiency: this approach is applied to known gene clusters in order to alter their expression and subsequently identify masked molecules in known isolates.

As stated earlier, most site-directed mutagenesis techniques, except CRISPR-BEST, involve recombination. Furthermore, recombination can use either internal systems of the cell, as in the PCR-mediated technique, or special enzymes: Cre-recombinase, Xisexcisionase, and Int-integrase. Undoubtedly, enzymes and specific enzyme-recognized sequences not only enhance precision, but also change the genomic content.

A number of site-directed mutagenesis techniques are based on the introduction of double-strand breaks, followed by DSB repair. These techniques include an I-SceI meganuclease-based system and CRISPR-Cas modifications (Cas9-based genome editing and Cpf1- assisted genome editing). All three approaches can be used to edit actinomycete BGCs. However, the genomic features of these bacteria impose a number of restrictions on the use of CRISPR-Cas9, given the off-target effect and toxicity of the Cas9 protein, and the restrictions on the use of I-SceI are due to the genome optimization associated with the generation of an 18-bp consensus meganuclease target sequence. The CRISPR-Cas system based on the Cas12 nuclease (Cpf1) recognizes a different T-rich PAM sequence, which reduces the risk of accidental double-strand breaks. In addition to the specific interaction between the nuclease and the target sequence, an important role is played by the internal cellular repair system associated with double-strand break repair.

Importantly, all these techniques require their own genetic constructs with the corresponding nucleotide sequences, which are used to transform streptomycete strains. A separate issue in all these approaches may be the low transformability of a particular strain. However, despite all the limitations of the described methodologies, they have allowed researchers to achieve good results–discovery of novel antibiotics and enhancement of the biosynthetic potential of actinomycetes. For example, in 2003, a PCR-mediated editing technique was used to perform manipulations with the geosmin cluster of a model *St. coelicolor* strain [36]. Seven years later, a Cre-recombinasebased technique was used to achieve a 1.4-Mb deletion in the geosmin cluster of a *St. avermitilis* strain [46]. When dealing with the site-specific approach, we should also mention the pSAM2 system. Despite the fact that this plasmid was generated back in 1989 [12], it was successfully applied in 2022 to introduce a mutation into the rifampicin cluster of *A. mediterranei DSM 40773 *cells [87]. An I-SceI meganuclease-based approach was used to produce a mutation in the actinorhodin cluster of* St. coelicolor A3(2) *cells in 2014 [30]. The possibility of using all the described techniques for genome manipulations has so far been demonstrated only in actinomycetes; CRISPR-Cas was the most effective approach that not only demonstrated a good outcome associated with the introduction of mutations, but also identified novel molecules. For example, using a CRISPR-Cas9-based technique, the ability of previously studied streptomycin-producing strains to synthesize the novel antibiotics thiolactomycin, phenanthroviridine, and 5-chloro-3-formylindole was revealed in 2019 [21].

Further prospects for the use of genome-editing techniques are associated with the opportunity to identify novel antibiotic BGCs in the genomes of characterized strains and induce targeted mutagenesis. This requires combining predictive bioinformatics algorithms for identifying potential BGCs of secondary metabolites and reliable tools for targeted mutations of regulatory sites, introduction of inducible promoters, and deletion of repressor genes. Transfer of target antibiotic BGCs into strains more suitable for expression seems promising. This approach may be especially effective for large-scale biotechnological production, when the production of a target metabolite is increased using a specially designed, genetically engineered strain, which will increase the profitability of the production.

## Abbreviations

BGC: biosynthetic gene cluster
PCR: polymerase chain reaction
NRP: nonribosomal peptide
PKS: polyketide synthase
UDG: Uracil-DNA glycosylase
DSB: double-strand break
